# Reference Genome Assembly for Australian *Ascochyta rabiei* Isolate ArME14

**DOI:** 10.1534/g3.120.401265

**Published:** 2020-04-28

**Authors:** Ramisah Mohd Shah, Angela H. Williams, James K. Hane, Julie A. Lawrence, Lina M. Farfan-Caceres, Johannes W. Debler, Richard P. Oliver, Robert C. Lee

**Affiliations:** * Centre for Crop and Disease Management, School of Molecular and Life Sciences, Curtin University, Bentley, WA, Australia,; ^†^ Murdoch University, Murdoch, WA, Australia, and; ^‡^ Department of Environment and Agriculture, Curtin University, Bentley, WA, Australia

**Keywords:** PacBio, *Pleosporales*, *Dothideomycetes*, plant pathogen, chickpea

## Abstract

*Ascochyta rabiei* is the causal organism of ascochyta blight of chickpea and is present in chickpea crops worldwide. Here we report the release of a high-quality PacBio genome assembly for the Australian *A. rabiei* isolate ArME14. We compare the ArME14 genome assembly with an Illumina assembly for Indian *A. rabiei* isolate, ArD2. The ArME14 assembly has gapless sequences for nine chromosomes with telomere sequences at both ends and 13 large contig sequences that extend to one telomere. The total length of the ArME14 assembly was 40,927,385 bp, which was 6.26 Mb longer than the ArD2 assembly. Division of the genome by OcculterCut into GC-balanced and AT-dominant segments reveals 21% of the genome contains gene-sparse, AT-rich isochores. Transposable elements and repetitive DNA sequences in the ArME14 assembly made up 15% of the genome. A total of 11,257 protein-coding genes were predicted compared with 10,596 for ArD2. Many of the predicted genes missing from the ArD2 assembly were in genomic regions adjacent to AT-rich sequence. We compared the complement of predicted transcription factors and secreted proteins for the two *A. rabiei* genome assemblies and found that the isolates contain almost the same set of proteins. The small number of differences could represent real differences in the gene complement between isolates or possibly result from the different sequencing methods used. Prediction pipelines were applied for carbohydrate-active enzymes, secondary metabolite clusters and putative protein effectors. We predict that ArME14 contains between 450 and 650 CAZymes, 39 putative protein effectors and 26 secondary metabolite clusters.

Ascochyta blight disease of chickpea (*Cicer arietinum* L.), is caused by *Ascochyta rabiei* (Pass.) Labrouse (teleomorph: *Didymella rabiei* (Kovachevski) von Arx, syn. *Mycosphaerella rabiei* (Kovachevski), syn *Phoma rabiei*) (de Gruyter *et al.* 2009; Aveskamp *et al.* 2010) and is the major biotic constraint affecting production of chickpea worldwide. Chickpea is a legume crop species of global economic importance (Fondevilla *et al.* 2015). Worldwide production was 12 M tonne in 2016 with two-thirds grown in India. Australia is a major exporter and the combined control costs and losses attributed to ascochyta blight in Australia in 2012 were around AU$40 M (Murray and Brennan 2012) which represents approximately 10% of crop value.

*A. rabiei* is a haploid, heterothallic, *Dothideomycete*s fungus (Order *Pleosporales*) and it causes disease by producing necrosis in leaf, stem and pod tissues (Trapero-Casas and Kaiser 1992; Peever *et al.* 2004). Little is known about how it causes disease. It produces solanapyrones, phytotoxic secondary metabolites in culture filtrates, and these have long been considered to play a role in pathogenicity (Alam *et al.* 1989; Hamid and Strange 2000). However deletion of the biosynthesis genes responsible for the synthesis of solanapyrones did not affect virulence (Kim *et al.* 2015a,b). Attention has since shifted to effectors, proteins that control the plant-pathogen interaction and in some cases induce necrosis (Lo Presti *et al.* 2015; Tan and Oliver 2017). In terms of pathogen life cycle and population structure, the two mating types are found in *A. rabiei* populations in Israel (Lichtenzveig *et al.* 2005), North America (Peever *et al.* 2004) and Canada (Armstrong *et al.* 2001), thus providing a mechanism for sexual recombination. In Australia, most reports suggest the presence of only mating-type MAT1-2 (Leo *et al.* 2015; Mehmood *et al.* 2017) and the absence of mating in the population. SSR genotyping has found the Australian *A. rabiei* population to be highly homogenous with around 70% of isolates tested being from a single dominant haplotype designated ARH01 (Leo *et al.* 2015; Mehmood *et al.* 2017).

In 2016, an Illumina short-read genome assembly of an Indian *A. rabiei* isolate, ArD2, was published (Verma *et al.* 2016). In their analysis Verma *et al.* (2016) predicted 758 secreted proteins from a total of 10,596 proteins encoded by the 34.6 Mb genome assembly (Verma *et al.* 2016). Of the 758 predicted secreted proteins, 201 proteins were annotated as Carbohydrate-Active Enzymes (CAZymes) (Lombard *et al.* 2014). These included proteins containing carbohydrate-binding modules and LysM domains that characterize chitin-binding effectors, which suppress the immune response of plants to fungal pathogens (de Jonge *et al.* 2010). There were 323 putative effectors with no known protein domain and 70 proteins of the predicted secretome showed sequence similarity to members of the Pathogen-Host Interaction (PHI) database (Winnenburg *et al.* 2006) with annotated involvement in virulence or pathogenicity (Verma *et al.* 2016). In addition to proposed virulence proteins, transcription factors that control gene expression during plant infection by *A. rabiei* have been predicted for ArD2 and assessed for their contribution to pathogenesis (Verma *et al.* 2017). Verma *et al.* (2017) suggest that for *A. rabiei*, Myb transcription factors play a role in regulating the expression of genes encoding secreted proteins, such as effectors. Gene regulation by transcription factors during plant infection may also vary depending on specific isolate and host interactions (Verma *et al.* 2017).

Here, we present a genome assembly for an Australian *A. rabiei* isolate, ArME14 that was produced by amalgamation of whole genome DNA sequence data from both Illumina and PacBio SMRT sequencing. The updated genome assembly features 12 full-length chromosome contigs with telomere sequences at both ends, 9 partial chromosomal contigs with one telomere end, and 13 smaller fragments with no telomere. The total assembled genome length was 40.9 Mb and genome annotation predicted 11,257 gene models.

## Materials and methods

### Fungal culture and DNA extraction

*A. rabiei* isolate ArME14 was collected from chickpea at the Department of Agriculture and Food Western Australia (DAFWA) field station, Medina, Western Australia in 2004. Fungal cultures were grown for three days in potato dextrose liquid media, at approx. 22° with shaking (150 rpm). For Illumina sequencing, DNA was prepared using a standard CTAB extraction method. RNA was removed by incubation with DNase-free RNase A and DNA was resuspended in TE buffer (10 mM Tris-HCl 1 mM EDTA, pH 8). DNA concentration was determined by NanoDrop Spectrophotometer (Thermo-Fisher Scientific, Waltham, MA, USA) and quality and purity were assessed by agarose gel electrophoresis. For PacBio sequencing, maxi-prep DNA extractions were produced using a modified method from Xin and Chen (2012). Fungal material was grown in Yeast Extract Glucose liquid media at approx. 22° with shaking (180 rpm), for 72 hr. DNA was resuspended in 2 mL Tris-HCl, pH 8.0 and treated with 20 µg.mL^-1^ DNase-free RNase A. DNA was purified using Ampure XP beads (Agencourt, Beckman-Coulter, USA) in a 96-well microtitre plate. The final DNA solution was quantified using Qubit and Nanodrop (Thermo Fisher Scientific) assays. Gel electrophoresis on a 1% agarose gel was used to assess DNA quality.

### Genome sequencing

Short-read DNA sequencing was performed at the Allan Wilson Genome Centre (Massey University, Palmerston North, New Zealand) using an Illumina Genome Analyzer (Illumina Inc., San Diego, CA, USA). Illumina TruSeq paired-end libraries were prepared for *A. rabiei* isolate ArME14 DNA, size-selected for 200 bp fragments, from which 75 bp reads were sequenced. Single-Molecule, Real-Time (SMRT) PacBio sequencing was performed by Genome Quebec (McGill University, Montreal, Canada). Libraries were prepared with size-selected 17 Kb fragments from sheared genomic DNA using P6-C4 chemistry, and sequenced on six SMRT cells using a PacBio RSII instrument (Pacific Biosciences, Menlo Park, CA, USA).

### Reference genome assembly

PacBio SMRT reads were assembled using the CANU v 1.2 assembler (Berlin *et al.* 2015) and the resulting intermediate assembly sequences were corrected using Illumina reads via PILON v 1.2.1 (Walker *et al.* 2014). A single mitochondrial genome contig from the assembly was identified by homology with other published *Dothideomycete* genome sequences and was designated ‘Mitochondrion MT’ in the new assembly. Sequencing statistics for the final corrected *A. rabiei* ArME14 reference assembly including overall percent GC content, were calculated using QUAST v 4.6.2 (Gurevich *et al.* 2013). Telomere sequences were manually recorded based on the presence of TTAGGG tandem repeat sequences at contig ends (Schechtman 1990). Computational steps were coordinated using Nextflow (Di Tommaso *et al.* 2017).

### Genome annotation and analysis

Gene prediction for the reference *A. rabiei* ArME14 assembly was performed using the annotation program, AUGUSTUS v 3.3 (Stanke *et al.* 2004, 2006; König *et al.* 2016), based on sequence homology of *in vitro* and *in planta* RNASeq and Massive Analysis of cDNA Ends (MACE) libraries from the *A. rabiei* BioProject, PRJNA288273 (Fondevilla *et al.* 2015). We used BUSCO version 3.0 (Simão *et al.* 2015) to assess assembly and annotation completeness by running protein fasta files with benchmarking against the Ascomycota_odb9 single-copy orthologs file downloaded from the BUSCO website September 2019 (https://busco.ezlab.org/). We used the program OcculterCut v 1.1 (Testa *et al.* 2016) to scan the genome assembly to determine its percent GC content distribution.

For detection and assessment of transposable element and repeat sequences, the suite of detection and classification programs in the PiRATE-Galaxy pipeline virtual machine as described by Berthelier *et al.* (Berthelier *et al.* 2018) was used. PiRATE-Galaxy uses similarity-based detection programs RepeatMasker (Smit *et al.* 2013) and TE-HMMER (Berthelier *et al.* 2018), a custom program based on HMMER (Eddy 1995) and tBLASTn (Altschul *et al.* 1990); structure-based programs MITE-Hunter (Han and Wessler 2010), SINE-Finder (Wenke *et al.* 2011), Helsearch (Yang and Bennetzen 2009) and LTRharvest (Ellinghaus *et al.* 2008); and repetitiveness-based programs, TEdenovo (Flutre *et al.* 2011) and RepeatScout (Price *et al.* 2005). CD-HIT-EST (Li and Godzik 2006) in PiRATE-Galaxy was used to reduce redundancy in the combined TE and repeat sequence dataset by removing duplicated sequences with 100% identity to other longer sequences in the data set. Short sequences of less than 500 nucleotides were removed. Classification of repeat and TE sequences was implemented using the program PASTEC (Hoede *et al.* 2014), using nucleotide, protein and profile HMM databanks from the PiRATE-Galaxy server (Berthelier *et al.* 2018).

Calculating coverage of ArME14 by the previous ArD2 assembly was performed with NUCMER (Kurtz *et al.* 2004) using the maxmatch argument and plotted using ggplot (Wickham 2016). Prediction of secreted proteins from the *A. rabiei* ArME14 set of annotated protein sequences was accomplished using SignalP version 5.0 (Almagro Armenteros *et al.* 2019), and DeepSig (Savojardo *et al.* 2018). From the set of annotated proteins, we applied effector selection criteria, including mature polypeptide molecular weight less than 25 KDa, number of cysteines, presence of a secretion signal and EffectorP 2.0 score greater than 0.8 (Sperschneider *et al.* 2016, 2018), to predict putative effector proteins using a custom pipeline written in Python [Johannes Debler. (2019, November 4). JWDebler/effector_selection: First working release (Version v1.0). Zenodo. http://doi.org/10.5281/zenodo.3526820]. Where there was disagreement between SignalP and DeepSig on the signal peptide processing site, the custom pipeline chose the site determined by SignalP.

CAZymes were identified from the ArME14 annotated set of proteins using the dbCAN2 web-based meta server (Zhang *et al.* 2018), implementing HMMER v 3.2.1 (Eddy 1996) with the HMMdb release 8.0, DIAMOND (Buchfink *et al.* 2015) and Hotpep (Busk *et al.* 2017). Secondary metabolite clusters were identified using the antiSMASH fungal version v 5.0, web-based prediction server (Medema *et al.* 2011; Blin *et al.* 2019). A CIRCOS plot that illustrates all annotated genome features was produced using the CIRCOS software v 0.69-9 (Krzywinski *et al.* 2009).

### Data availability

Illumina and PacBio genome sequencing data for *A. rabiei* ArME14 described herein, and the reference genome assembly have been deposited in the Sequence Read Archive and NCBI database, under the BioProject accession number PRJNA510692. *A. rabiei* ArME14 BioSample number is SAMN10613128. Illumina SRA entries are deposited under SRX5179494 and PacBio SRA data under SRX5172972. The GenBank assembly accession number is GCA_004011695.1. Supplemental material available at figshare: https://doi.org/10.25387/g3.11589420

## Results and discussion

**Figure 1:**
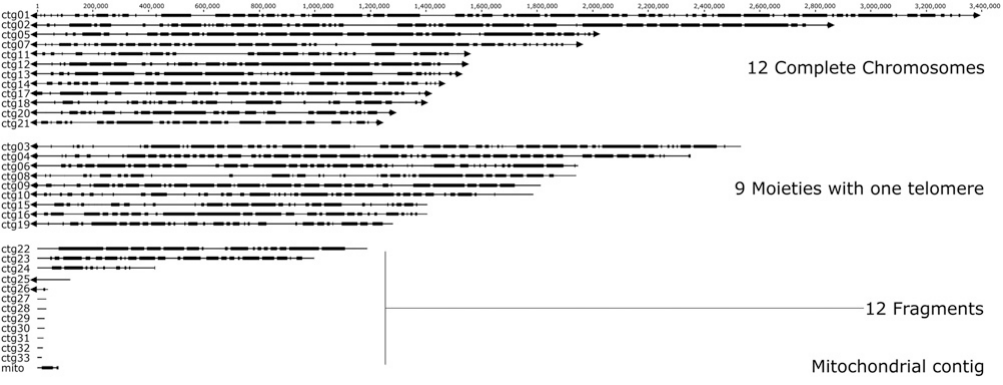
Genome contigs for the reference assembly of *A. rabiei* ArME14, produced from PacBio SMRT sequencing with polishing using Illumina sequencing. Nuclear contigs are labeled ctg01 to ctg33 as archived in NCBI BioProject PRJNA510692 and the mitochondrial contig is labeled mito. Gene-dense regions of the genome are shown as dark-shaded blocks, joined by gene-sparse and interspersed repeat-rich regions indicated by thin lines. Telomeres are indicated in the figure by triangles at the ends of respective contigs.

PacBio SMRT sequencing of ArME14 produced 34 contigs, including one mitochondrial genomic contig, at 166x sequencing depth (Table 1). The ArME14 assembly was 18% larger at 40,927,385 bp compared with 34,658,250 bp for the ArD2 genome assembly (Verma *et al.* 2016). Telomeres were identified manually by sequence observation and all were TTAGGG repeats of approximately 100 bp in length. Repeated TTAGGG sequences are reported to characterize the telomere regions in filamentous fungi such as *Neurospora crassa* (Schechtman 1990), *Cladosporium fulvum* (Coleman *et al.* 1993) and *Magnaporthe oryzae* (Rehmeyer *et al.* 2006; Farman 2007), and this sequence motif is a conserved feature in ArME14. Of the 33 nuclear contigs, 12 had TTAGGG telomere sequences at both ends (contig sizes 3,373,759 – 1,223,093 bp). Nine others had one telomere (contig sizes 2,532,578 – 1,278,587 bp). Values for L50 and N50 (Table 1) were 9 and 1,812,190 bp, compared with 64 and 154,808 bp for ArD2, respectively (Verma *et al.* 2016) (Figure 1). Akamatsu *et al.* (2012) used pulsed-field gel electrophoresis to determine chromosome number and size for multiple *A. rabiei* isolates from 21 countries. The number of chromosomes ranged from 12 to 16, and total genome size estimates ranged from 23 Mb to 34 Mb (Akamatsu *et al.* 2012). Our whole genome sequencing suggests that *A. rabiei* ArME14 possesses at least 17 chromosomes and has significantly higher genome size than previously estimated (Akamatsu *et al.* 2012). The mitochondrial genomic sequence was assembled as a single contig of 74,173 bp length (Figure 1) and was identified by homology with other fungal and *Dothideomycete* mitochondrial genome sequences. PacBio genome sequencing for fungi facilitates the assembly of long contigs by resolving the repetitive and AT-rich regions that characterize these species. For ArME14, we have been able to assemble 12 end-to-end chromosomal contigs as evidenced by the telomere sequences that terminate the contigs of the assembly. Recently produced, highly resolved genome assemblies for phytopathogenic fungi include: *Verticillium dahliae* (Faino *et al.* 2016), *Botrytis cinerea* (Van Kan *et al.* 2017), *Sclerotinia sclerotiorum* (Derbyshire *et al.* 2017) *Pyrenophora teres* (Syme *et al.* 2018) and *Pyrenophora tritici-repentis* (Moolhuijzen *et al.* 2018). For each of these PacBio sequencing was implemented but in addition to this, optical mapping, and in some cases genetic mapping were used to confirm the assembly, particularly across repetitive DNA within their genomes. For *V. dahliae*, optical mapping combined with PacBio sequencing improved the assembly from 119 contigs before optical mapping to 8 contigs after optical mapping (Faino *et al.* 2015). Even without optical and genetic mapping, but having deep sequencing coverage at 166x and Illumina correction, we are confident in the ArME14 genome sequence and the organization of GC-equilibrated and AT-rich sections in the assembly.

**Table 1:** Summary assembly and annotation statistics for Illumina sequencing of *A. rabiei* isolate, ArD2 (Verma *et al.* 2016) and PacBio SMRT sequencing for ArME14

Assembly statistics	ArD2 Illumina (13)	ArME14 PacBio SMRT
Genome size (bp)	34,658,250	40,927,385
Total sequenced bases	100 Gb	∼6.8 Gb
Coverage	178x	166x (928,353 reads)
Number of scaffolds/contigs	338 *^a^*	33 *^b^*
Largest scaffold/contig size (bp)	1,160,210 *^a^*	3,373,759 *^b^*
L50	64 *^a^*	9 *^b^*
N50 (bp)	154,808 *^a^*	1,812,190 *^b^*
GC (%)	51.6	49.2
% Repetitive sequence	9.9	12.6
Complete chromosomes	—	12
**Annotation statistics**		
Number of protein coding genes	10,596	11,257
Predicted secreted proteins	758 *^c^* (1,111 *^d^*)	1,145 *^c^*
Predicted effectors	328 *^c^* (36 *^d^*)	39 *^c^*
Predicted sec. metabolite clusters	26 *^e^*	26
Predicted no. of CAZymes	1,727 *^c^* (441 *^f^*)	451 *^c^* *^f^*

a Scaffolds for ArD2 Illumina assembly (GCA_001630375.1).

b Contigs for ArME14 PacBio SMRT assembly.

c Differences in numbers likely due largely to different selection criteria.

d Secretome and effector predictions for ArD2 assembly using the same methods applied to ArME14.

e Unknown prediction method for secondary metabolite clusters.

f CAZyme prediction using dbCAN2 meta server in this study.

**Figure 2:**
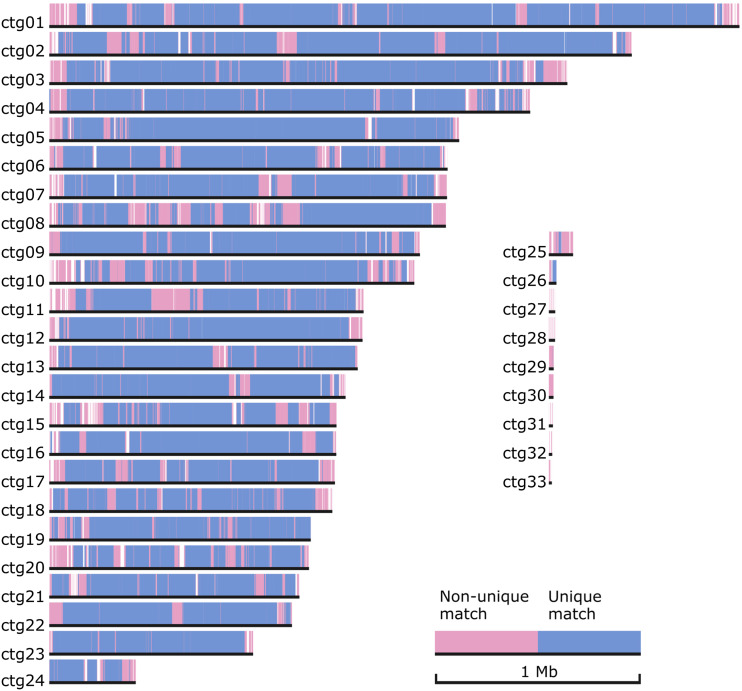
Alignment of *A. rabiei* ArD2 scaffolds to the 33 nuclear ArME14 contigs using NUCMER. Unique matches representing homologous nucleotide sequences between the two assemblies are indicated in blue, and repeat-rich nucleotide sequence that characterizes repetitive and AT-rich genomic regions are indicated by non-unique matches shown in red. Presumed non-assembled or absent sections from the ArD2 genome are represented by white space along each of the ArME14 reference contigs.

**Figure 3:**
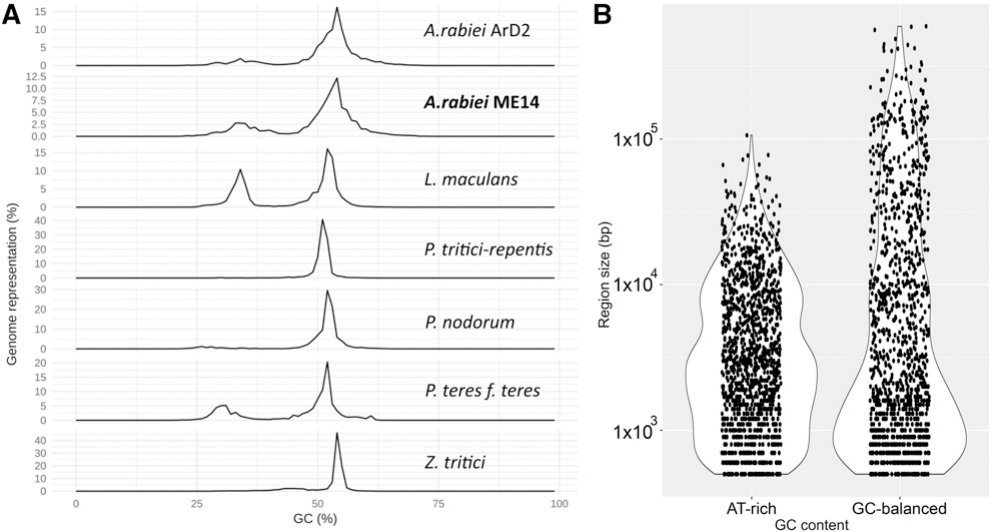
Summary of genome compartmentalisation into GC-equilibrated and AT-rich regions of the *A. rabiei* genome assemblies. (A) GC content (%) distribution for *Dothideomycete* genome assemblies as calculated by OcculterCut (Testa *et al.* 2016). (B) Size distributions of AT-rich and GC-balanced regions for *A. rabiei* ArME14 (note logarithmic scale).

AUGUSTUS (v 3.3) (Stanke *et al.* 2004, 2006; König *et al.* 2016) and expressed gene sequence data from a published *A. rabiei* (isolate P4) transcriptome project (Fondevilla *et al.* 2015) were used to annotate transcribed gene features of the ArME14 genome assembly. Figure 1 shows GC-balanced, gene-rich regions as thick black bars interspersed between AT-rich, gene-sparse regions. Homology-based alignment of the ArD2 and ArME14 genome assemblies (Figure 2) shows that unique and near-exact matches between the two genomes cover the majority of the ArME14 genome. However, a substantial proportion of the homologous regions are between non-uniquely matching sequences, which are likely to be repetitive regions in both assemblies. Prediction of transposable elements and other repetitive DNA sequences for ArME14 identified that these regions comprise approximately 6.14 Mb or 15% of the genome (Table 2) and this value is roughly equal to the amount of non-unique matches indicated by genome alignment (Figure 2). The most abundant of the transposable and repetitive element types present in the *A. rabiei* genome were the Class I, long terminal repeats (LTR) with 3.3 Mb (54%), Class II terminal inverted repeats (TIR), with 1.8 Mb (30%) and long interspersed nuclear element (LINE) with 0.45 Mb (7.3%). The ArME14 genome assembly had a lower overall GC content compared with the ArD2 assembly (Table 1). Using OcculterCut (Testa *et al.* 2016) we found that the content of AT-rich DNA sequence was higher for the ArME14 assembly than for the Illumina ArD2 assembly. Around 20% of the ArME14 genome has a low GC content (between 29% and 37% GC) compared to 9.4% for ArD2. The content of GC-equilibrated regions was 32 Mb for ArME14 and 30 Mb for ArD2. Overestimation of the amount of the repetitive DNA in the ArME14 genome assembly due to mis-assembly is possible, but the difference in AT-rich, repetitive DNA, between ArD2 and ArME14 can be explained by the more complete sequencing and assembly using PacBio sequencing. Distribution of GC content varies widely among the *Pleosporales* plant pathogenic fungi (Figure 3), with *Parastagonospora nodorum*, *Pyrenophora tritici-repentis* and *Zymoseptoria tritici* having mostly 50–55% GC content and the canola blackleg disease pathogen, *Leptosphaeria maculans*, having approximately one third of its genome as AT-rich DNA (Rouxel *et al.* 2011; Testa *et al.* 2016). *A. rabiei* has a similar GC content distribution to the barley pathogen, *Pyrenophora teres* f.sp *teres* (Figure 3 A). Size distributions for both the AT-rich and GC-balanced regions for ArME14 were highly variable, with average sizes of approximately 6,200 and 25,000 bp, respectively (Figure 3 B). Filamentous plant pathogen genomes tend to be characterized as having substantial proportions of repetitive and AT-rich sequence and their complement of genes includes a large number that encode secreted proteins. The genome architecture of ArME14 revealed by PacBio sequencing fits the “two-speed genome” model as proposed by Dong *et al.* (2015). The striking feature of this model, is that positive selection in genes located near repetitive DNA regions leads to higher rates of evolution in species for which genome architecture fits this model (Dong *et al.* 2015).

**Table 2:** Transposable element and repetitive DNA sequences from *A. rabiei* ArME14

Class	Type	Number of sequences	% of total sequences	Total nucleotides	% of total nucleotides	Average size
**I**	LTR	780	43	3336780	54	4278
	LINE	177	9.7	450418	7.3	2545
	LARD	26	1.4	194939	3.2	7498
	TRIM	5	0.3	5668	0.1	1134
	SINE	2	0.1	1034	0.02	517
**II**	TIR	683	38	1820841	30	2666
	Helitron	44	2.4	171920	2.8	3907
	MITE	7	0.4	4193	0.1	599
**SSR**	No cat *^a^*	82	4.5	137974	2.2	1682
	Host gene *^a^*	4	0.2	8223	0.1	2056
	SSR	6	0.3	5102	0.1	2056
**Total**		**1,816**	**100**	**6,137,092**	**100**	

a “No Cat” and “Host gene” are categories assigned by the PiRATE Galaxy server, and describe unclassified (no category) and potential host gene, respectively.

We predicted 11,257 protein coding genes in ArME14, 661 more than for ArD2, and again this discrepancy is likely a result of the different sequencing methods used and differences in the gene model prediction and annotation results. Using tBLASTn (Altschul *et al.* 1990), we identified 405 annotated ArME14 protein coding genes that were not found in the ArD2 genome assembly and almost all of these were located near contig ends or near annotated transposable element sequences. It is unclear whether the observed differences in the complement of genes between the two isolates is due to lack of sequence data for these regions in ArD2, difficulty in assembling such regions, or due to real deletions or insertions of gene-encoding sequence at these locations. Each of these possibilities can be explained by the AT-rich and repetitive nature of DNA sequence where these missing genes are located. BUSCO analysis indicated a substantial improvement for the sequencing and annotation for *A. rabiei* with only three missing and 12 fragmented genes for ArME14, compared with 37 missing and 17 fragmented for ArD2 (Supplementary Figure: Figure_S1).

Effector proteins of plant pathogenic fungi are usually predicted based on the presence of a secretion signal, small protein size and a high proportion of cysteine residues (Jones *et al.* 2018). Therefore our first step in effector protein prediction was to determine the set of secreted proteins. SignalP v 5.0 predicted 1,145 secreted proteins for ArME14. Verma *et al.* (2016) predicted fewer secreted proteins for ArD2 (758). However, when we applied the same prediction method for secreted proteins as for ArME14, we found a similar number (1,111) of secreted proteins for ArD2, suggesting that the two genomes are highly similar with respect to their complement of secreted proteins. We compared the 1,145 ArME14 secreted proteins with the 1,111 sequences predicted for ArD2 using tBLASTn and found that 22 ArD2 proteins were not present in the ArME14 genome and 29 proteins from ArME14 that were not in ArD2 (Supplementary File, File_S2). Table 3 shows the number of putative effector proteins predicted from the total *A. rabiei* ArME14 proteome using different selection criteria with increasing stringency. For ArD2, 328 effectors were predicted and these were a large proportion of the secreted, non-Carbohydrate Active Enzymes (Verma *et al.* 2016). In contrast, our study used EffectorP v 2.0 (Sperschneider *et al.* 2016, 2018) as a more specific tool for fungal effector prediction. Using a mature protein size threshold of 25 KDa and EffectorP score threshold of 0.8, we nominated 39 protein sequences, designated PE01 to PE39 as putative effectors (PE). Full details of the 39 putative effector proteins are presented in the Supplementary File, File_S4. Three of the ArME14 putative effectors were missing from the ArD2 proteome with only 36 ArD2 putative effectors being predicted using the same selection criteria as we used for ArME14. A subsequent tBLASTn search of the ArD2 assembly located one of these “missing” proteins, the ArME14 PE22 ortholog, as an un-annotated sequence in the ArD2 assembly. Putative effector genes PE 34 and PE36 in ArME14, were absent from the ArD2 nucleotide sequence. Both genes are located in highly repetitive regions of sub-telomeric DNA and may have been absent from ArD2 or not assembled correctly in the ArD2 Illumina genome assembly. From the set of ArD2 secreted proteins not found in ArME14, one was predicted to be an effector with mature protein molecular weight and EffectorP 2.0 score of 14.7 KDa and 0.64, respectively, although this was below our EffectorP threshold of 0.8. From the 29 ArME14 secreted proteins not in found ArD2, we predicted seven to be effectors with EffectorP score greater than 0.6, but only two having EffectorP scores above 0.8. These two proteins were PE34 and PE36 (Supplementary data) as discussed above. In the “two-speed genome” model, genes closely located to, or within highly repetitive sequence evolve at a higher rate with greater rates of positive selection (Oliver 2012; Grandaubert *et al.* 2014; Dong *et al.* 2015; Raffaele *et al.* 2015). This evolutionary process is illustrated in the case of seven small-secreted protein, avirulence effectors of *L. maculans*, where the corresponding genes are located in AT-rich regions of the *L. maculans* genome and display evidence of Repeat-Induced Point mutation (RIP) and positive selection in their sequences (Van de Wouw *et al.* 2010; Grandaubert *et al.* 2014). Similar evolutionary processes are likely to have shaped the pathogen-host relationship for *A. rabiei* and chickpea, and further insights about the molecular mechanisms of pathogenicity in this species will be uncovered through functional analysis of these predicted effector proteins.

**Table 3:** Summary of potential pathogenicity genome features, including: secondary metabolite clusters, predicted effector genes and CAZyme genes identified from the *A. rabiei* ArME14 genome assembly. Detailed tables are provided in Supplementary File, File_S4

Class	Number
**Putative effectors**	
EffP > 0.8, MW < 25KDa *^a^*	39
EffP > 0.8, MW < 15KDa *^a^*	27
EffP > 0.9, MW < 15KDa *^a^*	15
**Secondary metabolite clusters**	26
T1PKS	7
T3PKS	1
NRPS	2
NRPS-like	7
NRPS/NRPS-like – T1PKS	4
Indole	1
Terpene	4
**CAZymes**	451
AA - Auxiliary activities	77
CBM - Carbohydrate-binding module	3
CE - Carbohydrate esterase	31
GH - Glycoside hydrolase	227
GT - Glycosyl transferase	82
PL - Polysaccharide lyase	31

a mature protein MW.

The secondary metabolite cluster prediction tool, antiSMASH (Medema *et al.* 2011; Blin *et al.* 2019) predicted 26 clusters in both ArD2 and ArME14 (Table 3). Verma *et al.* (2016) similarly predicted 26 clusters for ArD2. The antiSMASH-predicted clusters in ArME14 matched clusters from the ArD2 genome assembly in almost all cases, with some BLAST hits spread across multiple ArD2 scaffolds. Notably, the NRPS/T1PKS cluster 10-1 on ArME14 contig 10 has a polyketide synthase gene (g5897) that was absent from the ArD2 assembly although other ortholog genes for the cluster were present. Details of the ArME14 secondary metabolite clusters are presented in the Supplementary File, File_S4. Predicted clusters were homologous to characterized clusters designated for the biosynthesis of known fungal secondary metabolites including: cluster 16.2, melanin (Akamatsu *et al.* 2010), cluster 3.1, mellein (Chooi *et al.* 2015), and cluster 7.3, solanapyrone (Kim *et al.* 2015a,b). There were a further six clusters with characterized secondary metabolite homologs with proposed roles in fungal physiology or reproduction and 17 other gene clusters putatively producing molecules with unknown structures and functions. It is likely that some of these gene clusters will have a role in producing novel molecules required for virulence and host specificity. Of the 26 secondary metabolite clusters, eight were located within sub-telomeric regions of the ArME14 assembly and two were bounded by highly repetitive regions populated by transposable elements. Similar to the predicted effectors, the presence of secondary metabolite clusters in repeat-rich regions of the genome confers mobility between species and rapid adaptation through processes such as Repeat-Induced Point mutation (RIP) (Hane and Oliver 2008; Fudal *et al.* 2009; Rouxel *et al.* 2011; Testa *et al.* 2016; Seidl and Thomma 2017). The features of the ArME14 genome are consistent with repetitive genome structure having played a role in the evolution and host adaptation of *A. rabiei*.

**Figure 4:**
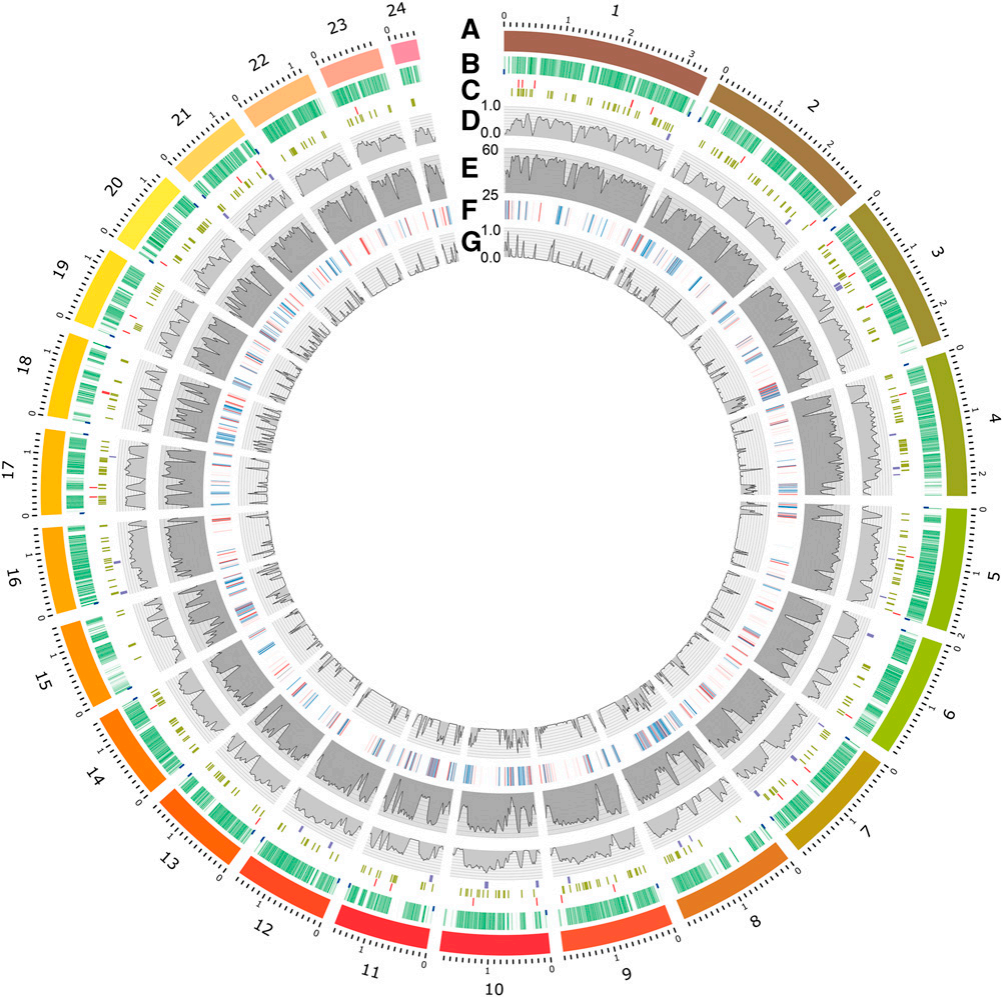
CIRCOS plot of key features of the *A. rabiei* ArME14 reference genome. Tracks labeled from outside: (A) The 26 largest contigs with size scale in Mb; (B) Annotated genes in scale (green) and telomeres (not to scale) (blue); (C) Locations (not to scale) of predicted effector genes (red), CAZyme genes (green), and predicted secondary metabolite clusters (purple and in scale); (D) Gene density with 20 Kb moving average window; (E) percent GC content with 50 Kb moving average window; (F) Transposable elements and repetitive DNA sequence regions. LINE, LTR, LARD, SINE and TRIM elements (blue), TIR, Helitron and MITE elements (red), SSRs (orange), No category, LTR/TIR, PLE/LARD and potential host gene (gray); (G) TE and repetitive DNA density with 20 Kb moving average window. An SVG version of this figure (Figure_S2) is included in the Supplementary Data to enable closer inspection of genome features.

Carbohydrate-Active Enzymes (CAZymes) are a key feature of all fungi, and in plant pathogens these enzymes are essential for the degradation of host plant polysaccharides for penetrating, colonizing and deriving nutrition from host tissues. Our CAZyme predictions from ArME14 produced 451 CAZyme sequences (Table 3), which is substantially fewer than the published number of 1,727 for ArD2 (Verma *et al.* 2016). Our search method identified only 441 CAZymes in ArD2. The dbCAN2 web server estimates of CAZyme number for *A. rabiei* are similar to those reported for other plant pathogenic fungi (Zhao *et al.* 2013). A total of 650 CAZymes were predicted by at least one of the tools and 451 to 650 is the likely range for the number of *A. rabiei* CAZymes. The main distinction of CAZyme complement among fungi is that necrotrophic fungal pathogens generally have a greater number (approx. 400-850) than biotrophs (approx. 170-320) (Zhao *et al.* 2013). The *A. rabiei* ArME14 genome has at least 450 and possibly up to 650 CAZymes, which is a similar number to those identified for other *Dothideomycete* genomes (Zhao *et al.* 2013). Fungal pathogens of dicots generally have an adapted set of CAZymes that are tailored to the types of carbohydrates found in dicot cell walls. Zhao *et al.* (Zhao *et al.* 2013) report that dicot pathogens generally have more polysaccharide lyases that degrade pectate and pectin (classes PL1 and PL3), which are more abundant in the cell walls of dicots than of monocots. In *A. rabiei* ArME14 there were nine PL1 CAZymes, which is similar to the average number reported for dicot pathogens and significantly greater than the average number of three PL1 enzymes for monocot pathogens (Zhao *et al.* 2013). In addition, *A. rabiei* ArME14 contained 12 pectin degrading polygalacturonases from GH28 where the average for dicot and monocot pathogens is 13 and 5, respectively (Zhao *et al.* 2013). Figure 4 summarizes the genome structure and locations of transposable and repetitive elements, putative effectors, CAZymes and secondary metabolite clusters in a CIRCOS plot. A plot of percent GC content in the CIRCOS format emphasizes partitioning of the genome into AT-rich gene-sparse, and GC-rich gene-dense sections. Notably 62% of the 39 predicted effector genes were located within 50 kb of repetitive regions and 23% were between 50-100 kb from the nearest repeat-rich region. Genome features are provided in a Supplementary genome feature file (Supplementary Data, File_S3).

Transcription factors are an important feature of the *A. rabiei* genome and of the 381 identified in the ArD2 genome assembly, three were found using tBLASTn searches to be absent from ArME14 (KZM27601.1, KZM27726.1 and KZM27745.1). Functional annotation of predicted ArME14 proteins using interproscan showed 126 proteins described as being transcription factors in addition to the 378 ArD2 transcription factor orthologs. Most of these were present in the ArD2 assembly but either they were not annotated as transcription factors or they were not annotated as protein-encoding genes for ArD2. We found three putative transcription factor genes in ArME14 for which there was no homologous DNA sequence in the ArD2 assembly. These were ArME14 g29, g427 and g4943, each described as containing fungal transcription factor domains. Putative transcription factor sequences from the comparisons between *A. rabiei* ArD2 and ArME14 are provided in the Supplementary Material, File_S2.

Developing an understanding of the mechanisms of virulence of plant pathogens is critical to the effective control of plant disease in crop production. The *Pleosporales* order of filamentous fungi including *P. nodorum*, *P. tritici-repentis*, *Cochliobolus heterostrophus* and *P. teres* f. *teres* among others, have many common overarching features that govern their primary functions as plant pathogens. Notwithstanding the similarities in genome structure and function among these species, there are also differences in virulence genes and effectors that determine the very important phenomenon of host specialization in plant pathogens. Furthermore, regulation of gene expression is critical to the production of virulence factors and the interaction of pathogen and plant host (Verma *et al.* 2017). The publication of a near-complete, high-fidelity genome assembly for *A. rabiei* complements the previously published genome assembly (Verma *et al.* 2016) and provides the basis for further work in the field of chickpea ascochyta blight research.
